# Diagnostic Value of Clinical Features to Distinguish Enteric Fever From Other Febrile Illnesses in Bangladesh, Nepal, and Pakistan

**DOI:** 10.1093/cid/ciaa1297

**Published:** 2020-12-01

**Authors:** Kristen Aiemjoy, Dipesh Tamrakar, Shampa Saha, Shiva R Naga, Alexander T Yu, Ashley Longley, Kashmira Date, Caitlin Hemlock, Farah N Qamar, Samir K Saha, Stephen P Luby, Denise O Garrett, Jason R Andrews, Isaac I Bogoch

**Affiliations:** 1 Division of Infectious Diseases and Geographic Medicine, Stanford University School of Medicine, Stanford, California, USA; 2 Dhulikhel Hospital, Kathmandu University Hospital, Dhulikhel, Nepal; 3 Child Health Research Foundation, Department of Microbiology, Dhaka Shishu Hospital, Dhaka, Bangladesh; 4 National Foundation for the Centers for Disease Control and Prevention, Atlanta, Georgia, USA; 5 Global Immunization Division, Centers for Disease Control and Prevention, Atlanta, Georgia, USA; 6 Applied Epidemiology, Sabin Vaccine Institute, Washington, DC, USA; 7 Department of Pediatrics and Child Health, Aga Khan University Karachi, Pakistan; 8 Bangladesh Institute of Child Health, Dhaka Shishu Hospital, Sher-E-Bangla Nagar, Dhaka, Bangladesh; 9 Department of Medicine, University of Toronto, Toronto, Ontario, Canada

**Keywords:** Enteric fever, typhoid, South Asia, clinical diagnosis

## Abstract

**Background:**

Enteric fever, a bacterial infection caused by *Salmonella enterica* serotypes Typhi and Paratyphi A, frequently presents as a nonlocalizing febrile illness that is difficult to distinguish from other infectious causes of fever. Blood culture is not widely available in endemic settings and, even when available, results can take up to 5 days. We evaluated the diagnostic performance of clinical features, including both reported symptoms and clinical signs, of enteric fever among patients participating in the Surveillance for Enteric Fever in Asia Project (SEAP), a 3-year surveillance study in Bangladesh, Nepal, and Pakistan.

**Methods:**

Outpatients presenting with ≥3 consecutive days of reported fever and inpatients with clinically suspected enteric fever from all 6 SEAP study hospitals were eligible to participate. We evaluated the diagnostic performance of select clinical features against blood culture results among outpatients using mixed-effect regression models with a random effect for study site hospital. We also compared the clinical features of *S.* Typhi to *S.* Paratyphi A among both outpatients and inpatients.

**Results:**

We enrolled 20 899 outpatients, of whom 2116 (10.1%) had positive blood cultures for *S.* Typhi and 297 (1.4%) had positive cultures for *S.* Paratyphi A. The sensitivity of absence of cough was the highest among all evaluated features, at 65.5% (95% confidence interval [CI], 55.0–74.7), followed by measured fever at presentation at 59.0% (95% CI, 51.6–65.9) and being unable to complete normal activities for 3 or more days at 51.0% (95% CI, 23.8–77.6). A combined case definition of 3 or more consecutive days of reported fever and 1 or more of the following (a) either the absence of cough, (b) fever at presentation, or (c) 3 or more consecutive days of being unable to conduct usual activity--yielded a sensitivity of 94.6% (95% CI, 93.4–95.5) and specificity of 13.6% (95% CI, 9.8–17.5).

**Conclusions:**

Clinical features do not accurately distinguish blood culture–confirmed enteric fever from other febrile syndromes. Rapid, affordable, and accurate diagnostics are urgently needed, particularly in settings with limited or no blood culture capacity.

Enteric fever is a systemic bacterial infection caused by *Salmonella enterica* subspecies enterica serotypes Typhi and Paratyphi. Estimates of enteric fever incidence range from 550 per 100 000 in South Asia to 160 per 100 000 in Western sub-Saharan Africa to 0.3 per 100 000 in Western Europe [1].

Typhoid and Paratyphoid commonly cause nonlocalizing febrile illnesses that present similarly to many other bacterial, viral, and parasitic fevers. Physicians have struggled to distinguish enteric fever from other fever etiologies for hundreds of years, potentially even as early as the era of Hippocrates [2–4]. While the advent of bacterial culturing techniques in the early 20^th^ century improved the accuracy of diagnosing enteric fever, the cost and logistical hurdles of setting up microbiology facilities put them largely out of reach for many of the regions with the highest enteric fever burden [5]. Even when blood culture capacity is available, testing can be costly, has limited sensitivity, and is slow, with results taking up to 5 days [6]. For these reasons, physicians managing patients with enteric fever in low- and middle-income countries often rely on clinical features, including both reported symptoms and clinical signs to make diagnoses and choose treatment regimens.

Despite the reliance of clinicians on clinical features to diagnose enteric fever, accurate clinical prediction rules have remained elusive [7–9]. We evaluated the diagnostic performance of clinical features of blood culture–confirmed *S.* Typhi and *S.* Paratyphi A patients enrolled in the Surveillance of Enteric Fever in Asia Project (SEAP), conducted in Bangladesh, Nepal, and Pakistan. We also compared the clinical features of *S.* Typhi patients to *S.* Paratyphi A patients. Our objectives were to identify those clinical features that might have diagnostic value for identifying enteric fever and that might distinguish *S.* Paratyphi A from *S.* Typhi.

## METHODS

### Study Design Overview

SEAP was a prospective surveillance study for enteric fever in Bangladesh, Nepal, and Pakistan. Participants were enrolled from September 2016 through September 2019. In each country, there were 2 SEAP enrollment hospitals; the facility coverage was urban for Bangladesh (Dhaka) and Pakistan (Karachi), and both urban (Kathmandu) and peri-urban (Kavre) in Nepal. Participants were enrolled from outpatient and inpatient departments, as well as from the hospital laboratory. Both study sites in Bangladesh served a primarily pediatric population.

### Study Population

To evaluate those clinical symptoms that might distinguish enteric fever (*S*. Typhi or *S*. Paratyphi) from other febrile illnesses, we included all SEAP participants recruited from outpatient departments. Outpatient department participants were individuals living in the predefined catchment area of the hospital who presented to the study facility outpatient department with 3 or more consecutive days of fever and received a blood culture [10].

To compare the clinical features of *S.* Typhi to *S.* Paratyphi, we included SEAP participants recruited from the inpatient and outpatient departments, and from the hospital laboratory. For this analysis, we included individuals who presented to the outpatient department with 3 or more consecutive days of reported fever and received a blood culture; individuals presenting to the inpatient department with clinically suspected enteric fever, with or without blood culture; or individuals presenting to the inpatient department or hospital laboratory with a blood culture–confirmed *S.* Typhi or *S.* Paratyphi infection. Individuals presenting to outpatient departments were only eligible if they resided within the predefined catchment area, whereas individuals presenting to inpatient departments or the hospital lab were eligible regardless of their residential address. The days of fever criteria were measured by self-report or caregiver-report.

### Measurements

A sample of peripheral venous blood was collected from each study participant in either a BD BACTEC (TM) or BD BACTEC PEDS Plus (TM) aerobic bottle and incubated for up to 5 days, using the BACTEC automated culture system (BACTEC; Becton Dickinson, Baltimore, MD). Indicator-positive samples were then subcultured onto MacConkey agar plates and nonselective media (sheep blood agar). Species were confirmed using biochemical testing and O and H antisera (BD Laboratories), if available.

Research assistants reviewed hospital records and entered information on physical exam findings and additional laboratory test results into a custom-built, electronic data-capture system. Complete blood count results were only available from participants for whom the test was indicated and performed. Temperature was assessed on arrival to the outpatient department or upon admission to the inpatient department. Fever start date, days of being unable to conduct normal activity, cough, diarrhea, constipation, abdominal pain, vomiting, nausea, and headache were all measured using self-r or caregiver-report and were collected directly from the patient or guardian at the time of enrollment into SEAP. We also recorded the clinician’s assessment of whether the patient had enteric fever, which was made before blood culture results were available.

### Statistical Analyses

We evaluated associations and calculated the diagnostic performance of clinical features using mixed-effect logit models with a random effect for study site hospital, adjusted for age. Sensitivity and specificity were calculated as the probability of the dichotomous result of the index test (ie, clinical feature), conditional on the blood culture being positive (sensitivity) or negative (specificity). The positive predictive value (PPV) and negative predictive value were calculated as the probability of blood culture positivity, conditional on the index test being positive or negative, respectively. We estimated probabilities and standard errors from the logit models using the emmeans package (version 1.4.4) in R. We evaluated the top 2 and top 4 performing symptoms in combination (performance defined by the sensitivity). We also assessed the validity of the clinician’s suspected diagnosis of enteric fever, using the same approach both overall and by country.

To compare gastrointestinal symptoms among patients based on the blood culture outcome and age strata, we used generalized estimating equations that accounted for clustering by study site hospital.

To evaluate differences in the clinical features of *S.* Typhi and *S.* Paratyphi infections, we used mixed-effect regression models with a random effect for study site hospital. All variables were evaluated in independent models and were adjusted for age (with the exception of age itself, gender, and recruitment site). All analyses were performed in R studio (R version 3.6.0).

### Ethics Statement

Written informed consent was obtained from all participants. For minors, verbal assent was provided in addition to informed consent from a parent or guardian. The study was approved by the Bangladesh Institute of Child Health Ethical Review Committee, Nepal Health Research Council, Aga Khan University Ethical Review Committee, National Bioethics Committee of Pakistan, Institutional Review Board at Stanford University, and US Centers for Disease Control and Prevention.

## RESULTS

### Enteric Fever Clinical Features Among Patients Presenting to Outpatient Departments with Three or More Consecutive Days of Reported Fever

Over the 3-year study period, we enrolled 20 899 patients with ≥3 days of reported fever from outpatient departments. All patients received a blood culture, yielding 2116 (10.1%) *S.* Typhi, 297 (1.4%) *S.* Paratyphi A, and 18 486 (88%) negative cultures (Table 1). The median age of enteric fever outpatients was 6 years (5 in Bangladesh, 19 in Nepal, and 6 in Pakistan), compared with 5 years among non–enteric fever patients (3 in Bangladesh, 19 in Nepal, and 8 in Pakistan).

**Table 1.** Demographic and clinical characteristics of outpatients with reported fever ≥3 days, by enteric fever blood culture result -- Bangladesh, Nepal, and Pakistan, 2016-2019

	Enteric Fever (+), n = 2413	Enteric Fever (−), n = 18 486
Female	1057 (43.8%)	8105 (43.8%)
Age, years		
Median (IQR)	6 (3–10)	5 (2–14)
Febrile at presentation, >99.5°F	1321 (58.5%)	7437 (44.4%)
High-grade fever at presentation, ≥103°F	109 (4.8%)	421 (2.5%)
Temperature at presentation, °F		
Median (IQR)	100 (98.6–101.0)	99.2 (98.1–100.4)
Days of fever		
Mean (SD)	5.6 (3.1)	5.2 (3.0)
Days unable to conduct usual activity		
Mean (SD)	3.1 (3.3)	2.7 (3.0)
Cough	722 (29.9%)	9623 (52.1%)
Diarrhea	386 (16.0%)	2159 (11.7%)
Constipation	135 (5.6%)	1289 (7.0%)
Abdominal pain	626 (26.0%)	3677 (20.1%)
Vomiting	760 (31.5%)	5095 (27.6%)
Nausea	14 (.6%)	282 (1.5%)
Headache	620 (25.9%)	5581 (30.8%)
Leukopenia	72 (9.2%)	710 (13.0%)
Thrombocytopenia	70 (8.9%)	628 (11.5%)
Diagnosed with enteric fever	1678 (69.6%)	7961 (43.1%)

Data are of outpatients presenting with 3 or more consecutive days of fever to SEAP study site facilities Bangladesh, Nepal, and Pakistan based on their enteric fever (Salmonella Typhi or Salmonella Paratyphi) blood culture result.

Abbreviations: IQR, interquartile range; SD, standard deviation.

**Figure 1.**
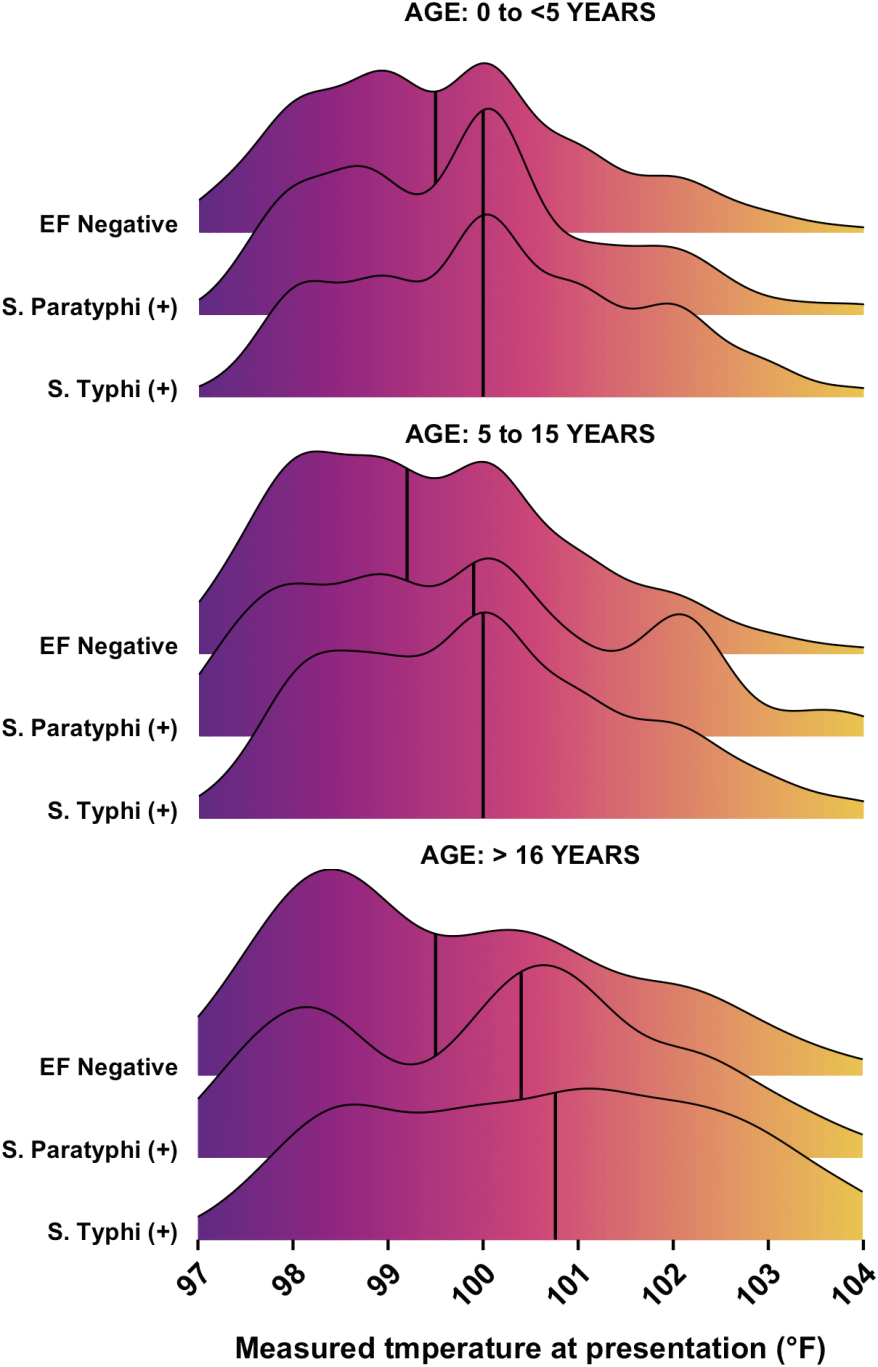
Distribution of temperature at presentation among 20 899 outpatients presenting with 3 or more consecutive days of reported fever to SEAP study site hospitals in Bangladesh, Nepal, and Pakistan. The solid black vertical lines indicate the median intake temperature. Abbreviation: EF negative, blood culture negative for enteric fever; SEAP, Surveillance for Enteric Fever in Asia Project.

At presentation to the outpatient department, 1321 (59%) enteric fever patients were febrile (>99.5°F), compared with 7437 (44%) blood culture–negative febrile patients; 109 (4.8%) of the enteric fever patients had a high-grade fever (≥103°F), compared with 421 (2.5%) of the blood culture–negative febrile patients (Table 1). The median temperature upon presentation to the outpatient department was higher among both *S.* Typhi and *S.* Paratyphi A patients than among non–enteric fever patients across all age groups (Figure 1). The mean temperature upon presentation was 100.7 °F for patients who were blood culture positive for enteric fever and 99.6 °F for patients who were blood culture negative for enteric fever (*P* < .00001)

**Figure 2.**
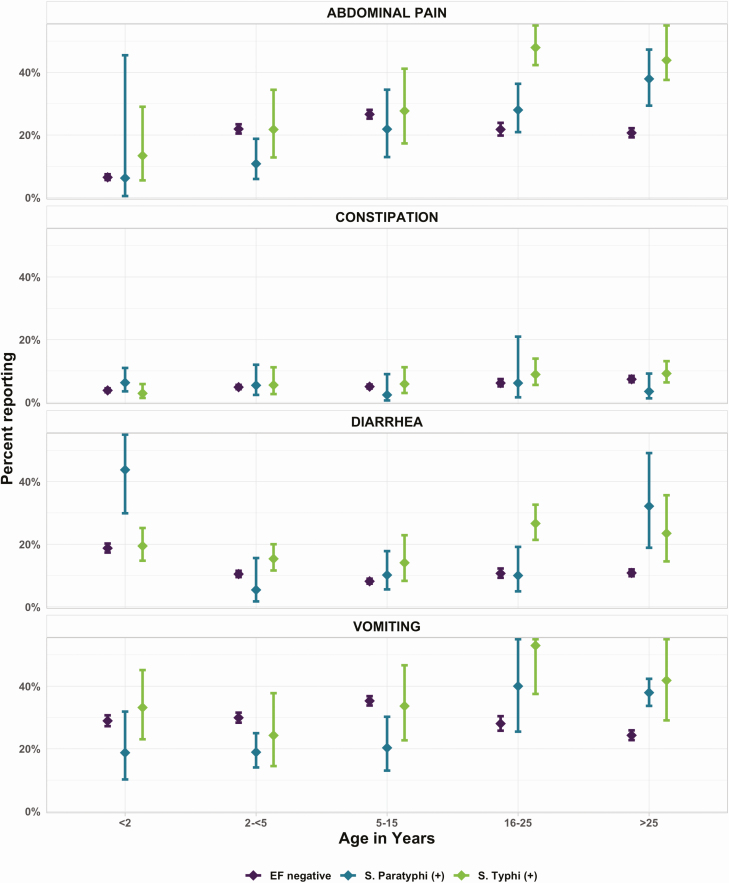
Gastrointestinal symptoms among outpatients with 3 or more consecutive days of fever to SEAP study site hospitals in Bangladesh, Nepal, and Pakistan. Point estimates and confidence intervals were calculated using generalized estimating equations to account for clustering by study site hospital. Abbreviations: EF negative, blood culture negative for enteric fever; SEAP, Surveillance for Enteric Fever in Asia Project.

**Figure 3.**
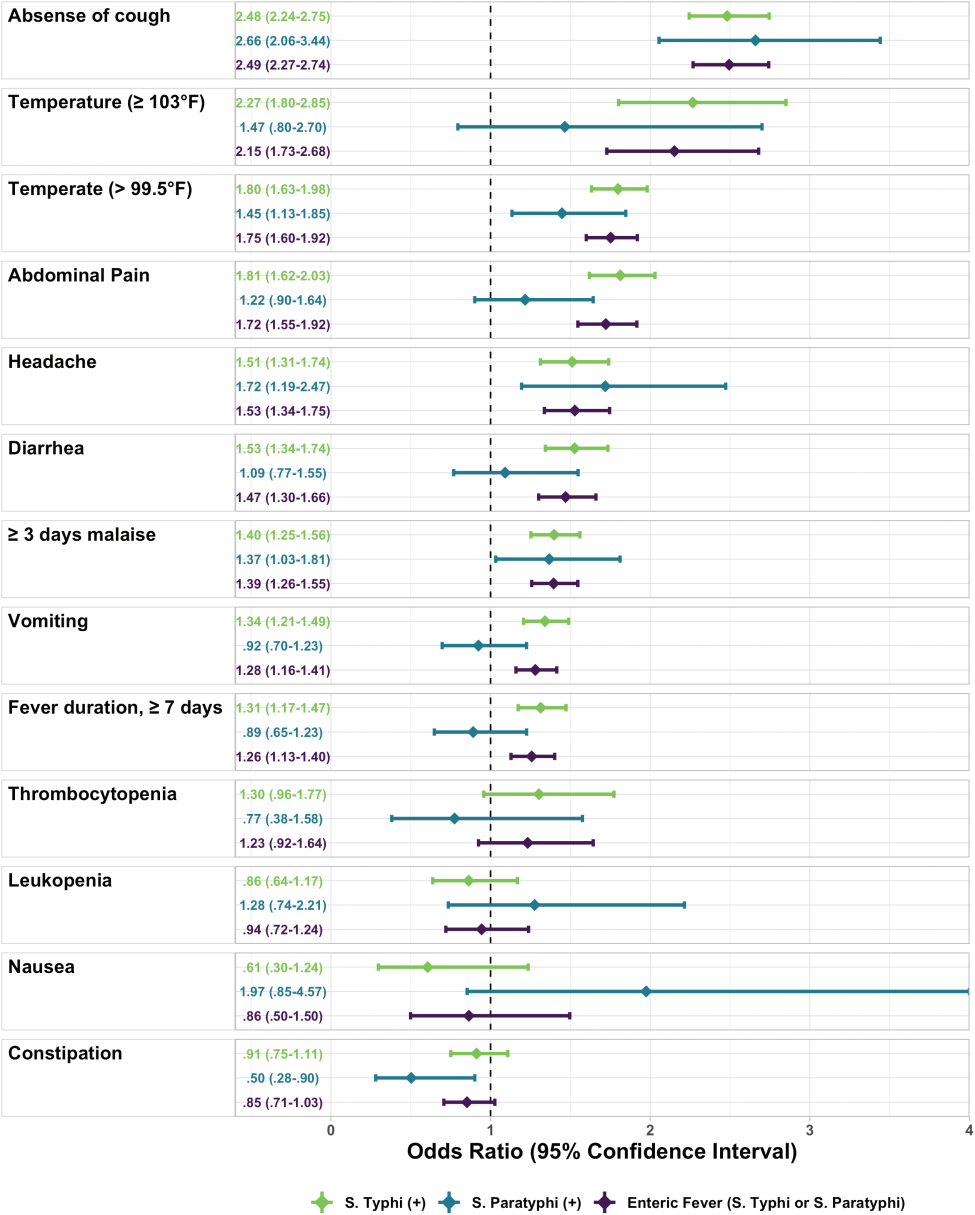
Comparison of the characteristics of *Salmonella* Typhi, *Salmonella* Paratyphi, and enteric fever (Salmonella Typhi or Salmonella Paratyphi) among 20 899 outpatients presenting with 3 or more consecutive days of fever to SEAP study site hospitals in Bangladesh, Nepal, and Pakistan. Odds ratios and 95% confidence intervals were estimated using mixed-effect logistic regression models with a random effect for study site hospital. Abbreviation: SEAP, Surveillance for Enteric Fever in Asia Project.

The most commonly reported symptoms were similar among enteric fever patients and enteric fever culture-negative patients, though enteric fever patients were less likely to report cough or headache than culture-negative febrile patients, but more likely to report abdominal pain, vomiting, or diarrhea (Table 1). The presentation of abdominal pain, diarrhea, and vomiting among enteric fever patients was age dependent. Adults (>25 years) with enteric fever were more likely to report abdominal pain than febrile adults without enteric fever. Diarrhea was more common in young children (<2 years) with *S.* Paratyphi A, and among adults (>25 years) with *S.* Typhi and *S.* Paratyphi A. Constipation did not have an age-dependent relationship (Figure 2). Patients in Nepal and Pakistan were more likely to report cough, abdominal pain, and headache as compared to patients in Bangladesh, even after accounting for the age differences in the populations (Table 2). In the age-adjusted analysis, fever (>99.5o F) at presentation, duration of fever ≥7 days, headache, absence of cough, and inability to conduct usual activities ≥3 days, as well as abdominal pain, diarrhea and vomiting were associated with enteric fever (Figure 3).

**Table 2.** Characteristics of outpatients aged ≤15 years with blood-culture confirmed enteric fever, by study country — Bangladesh, Nepal, and Pakistan, 2016–2019

	Bangladesh, n = 1448	Nepal, n = 77	Pakistan, n = 542
Female	665 (45.9%)	28 (36.4%)	240 (44.3%)
Age in years			
Mean (SD)	5.2 (2.9)	9.1 (3.7)	5.2 (3.8)
Febrile at presentation, >99.5°F	802 (57.2%)	28 (54.9%)	314 (58.4%)
High-grade fever at presentation, ≥103°F	59 (4.2%)	1 (2.0%)	20 (3.7%)
Temperature at presentation, °F			
Mean (SD)	99.8 (1.7)	99.7 (2.0)	100.0 (1.7)
Days of fever			
Mean (SD)	5.3 (2.7)	5.4 (3.0)	6.2 (3.6)
Days unable to conduct usual activity			
Mean (SD)	2.1 (2.6)	4.3 (3.1)	4.2 (3.6)
Cough	299 (20.7%)	34 (44.2%)	242 (44.6%)
Diarrhea	169 (11.7%)	15 (19.5%)	120 (22.2%)
Constipation	72 (5.0%)	2 (2.6%)	33 (6.1%)
Abdominal pain	211 (14.6%)	37 (48.1%)	229 (42.6%)
Vomiting	293 (20.2%)	33 (42.9%)	273 (50.4%)
Nausea	1 (.1%)	3 (3.9%)	1 (.2%)
Headache	73 (5.0%)	43 (57.3%)	202 (38.3%)
Leukopenia	10 (2.4%)	3 (7.1%)	19 (13.1%)
Thrombocytopenia	7 (1.7%)	4 (9.5%)	21 (14.5%)
Diagnosed with enteric fever	1127 (77.8%)	42 (54.5%)	359 (66.2%)

Data are from outpatient children 15 years and younger with blood culture–confirmed enteric fever.

Abbreviation: SD, standard deviation.

The sensitivity and specificity of using fever (>99.5°F) at presentation as a diagnostic criterion were 59.0% (95% confidence interval [CI], 51.6–65.9) and 55.9% (95% CI, 48.6–63.3), respectively; the PPV was 13.1% (95% CI, 9.3–18.2; Table 4). The sensitivity of absence of cough was the highest among all the clinical features evaluated, at 65.5% (95% CI, 55.0–74.7), followed by fever (>99.5°F) at presentation at 59.0% (95% CI, 51.6–65.9), and then by having 3 or more days of being unable to conduct activity at 51.0% (95% CI, 23.8–77.56). Even though abdominal pain, headache, diarrhea, vomiting, and duration of fever (≥7 days) were all positively associated with enteric fever in the age-adjusted analysis (Figure 3), the sensitivities for these symptoms did not surpass 35%. For the combined case definition of 3 or more consecutive days of reported fever and one or more of the following -- (a) absence of cough, (b) fever at presentation, (c) 3 or more days of being unable to conduct usual activity, the sensitivity was 94.6% (95% CI, 93.4–95.5) and the specificity was 13.6% (95% CI, 9.8–17.5). The PPV was just 10.5% (95% CI, 7.2–15.0), and did not exceed the enteric fever blood culture–positive prevalence in either Pakistan (14.3%) or Bangladesh (13.2%; Table 3).

**Table 3.** Performance of clinical symptoms for diagnosing enteric fever among outpatients with reported fever ≥3 days — Bangladesh, Nepal, and Pakistan, 2016–2019

	Sensitivity (95% CI)	Specificity (95% CI)	PPV^a^ (95% CI)	NPV^a^ (95% CI)
Clinical signs				
Febrile at presentation, >99.5°F	59.0% (51.6–65.9)	55.9% (48.6–63.3)	13.1% (9.3–18.2)	92.6% (89.4–95.8)
High-grade fever at presentation, ≥103°F	5.0% (3.5–7.3)	97.6% (96.4–98.9)	20.5% (16.5–25.1)	90.4% (86.5–94.4)
Leukopenia	6.3% (3.3–11.6)	88.2% (84.4–92.1)	7.9% (5.0–12.1)	84.5% (69.2–99.7)
Thrombocytopenia	7.0% (2.8–16.7)	89.5% (82.3–96.6)	7.6% (4.1–13.9)	84.8% (69.1–100.6)
Symptoms				
Absence of cough	65.5% (55.0–74.7)	54.7% (43.5–65.9)	14.2% (10.5–19.0)	93.5% (90.7–96.3)
≥3 days of being unable to conduct activity	51.0% (23.8–77.6)	57.1% (27.3–86.9)	10.5% (7.6–14.4)	91.8% (87.4–96.1)
Vomiting	35.2% (23.3–49.2)	69.8% (54.9–84.8)	11.3% (7.6–16.5)	90.5% (86.8–94.2)
Headache	33.4% (11.8–65.3)	71.8% (43.5–100.1)	9.1% (6.4–13.0)	91.7% (86.6–96.7)
Abdominal pain	29.5% (17.3–45.4)	80.0% (66.9–93.0)	12.8% (8.6–18.8)	90.9% (87.5–94.4)
Fever duration, ≥7 days	24.0% (20.1–28.4)	80.0% (77.5–82.5)	12.7% (9.3–17.3)	89.4% (85.6–93.2)
Diarrhea	16.8% (10.4–26.1)	88.4% (82.8–94.1)	13.6% (9.8–18.6)	90.5% (86.8–94.2)
Constipation	4.1% (1.7–9.5)	94.9% (91.1–98.7)	8.1% (5.8–11.1)	89.9% (86.0–93.7)
Combined case definition				
≥ 3 days of reported fever AND either a) absence of cough OR b) fever at presentation (>99.5 °F)	88.0% (83.8–91.1)	30.1% (19.9–40.2)	12.6% (9.0–17.2)	95.6% (93.2–98.1)
≥ 3 days of reported fever AND a) Absence of cough OR b) measured fever at presentation (>99.5 °F) OR c) ≥ 3 days of being unable to conduct activity	94.6% (93.4–95.5)	13.6% (9.8–17.5)	10.5% (7.2–15.0)	97.0% (94.1–100.0)
Multiple symptoms: febrile at presentation, absence of cough, ≥3 days of being unable to conduct activity, vomiting, headache, abdominal pain				
Any 2 of above symptoms	88.0% (77.5–94.0)	27.4% (17.3–37.6)	11.5% (8.0–16.2)	96.1% (92.8–99.4)
Any 3 of above symptoms	55.5% (37.9–71.8)	64.2% (46.6–81.8)	13.5% (9.6–18.7)	93.5% (89.5–97.5)
Any 4 of above symptoms	23.4% (10.8–43.3)	87.3% (76.1–98.4)	14.1% (10.3–18.9)	91.6% (87.5–95.6)

Sensitivity, specificity, PPV, and NPV were calculated using mixed-effects logit models with a random effect for study hospital, and were adjusted for age.

Abbreviations: NPV, negative predictive value; PPV, positive predictive value.

^a^The PPV and NPV rely on the underlying prevalence of disease. In this population, the prevalence of blood culture–positive enteric fever was 11.6% (2416/20 899), with prevalences of 13.2% (1453/10 990) in Bangladesh, 5.3% (266/5047) in Nepal, and 14.3% (697/4862) in Pakistan.

### Comparison of *Salmonella* Typhi and Paratyphi A Clinical Signs and Symptoms

In addition to outpatients, we enrolled *S*. Typhi and *S*. Paratyphi A blood culture–positive patients from inpatient departments and the hospital lab, resulting in a total of 4610 patients with *S.* Typhi and 605 with *S.* Paratyphi A. Overall, *S.* Paratyphi A comprised 11.6% (605/ 5215) of enteric fever cases, including 12.8% (387/3032) in Bangladesh, 19.3% (103/533) in Nepal, and 7.0% (115/1650) in Pakistan.

Individuals with microbiologically confirmed S. Typhi were younger than S. Paratyphi A patients (mean age 11.5 versus 13.5 years, p<0.001). Patients with *S*. Typhi were more likely to be admitted to an inpatient department than those with *S*. Paratyphi A (20.8% vs 12.7%, respectively; *P* < .001); and *S.* Typhi patients were more likely to be both febrile (>99.5°F) and present with a high-grade fever (≥103°F) at their initial clinical presentation compared with patients with *S.* Paratyphi A. Abdominal pain, diarrhea and vomiting were more frequently reported by patients with *S.* Typhi than those with *S.* Paratyphi A (Table 4).

**Table 4.** Comparison of Salmonella enterica serovar Typhi and S. enterica serovar Paratyphi A among patients with culture-confirmed enteric fever — Bangladesh, Nepal, and Pakistan, 2016–2019

	*S.* Tyhpi, n = 4610	*S.* Paratyhpi A, n = 605	*P* value
Age in years	11.5 (5.6–17.4)	13.5 (7.7–19.4)	<.001
Female	43.3% (41.1–45.5)	42.1% (37.9–46.4)	.582
Inpatient	20.8% (11.0–35.8)	12.7% (6.2–24.1)	<.001
Fever duration in days^a^	7.6 (6.3–8.8)	6.9 (5.7–8.2)	.002
Temperature at presentation, °F^a^	100.8 (100.5–101.1)	100.2 (99.8–100.5)	<.001
Febrile at presentation/admission, >99.5°F^a^	70.4% (64.6–75.6)	61.6% (53.9–68.8)	<.001
High-grade fever at presentation/admission, ≥103°F^a^	15.1% (10.0–22.1)	8.7% (5.2–14.0)	<.001
Days of being unable to conduct activity^a^	4.6 (2.6–6.6)	4.0 (2.0–6.0)	<.001
Antibiotics taken prior to presentation/admission	54.1% (44.6–63.3)	47.2% (37.2–57.4)	.002
Cough^a^	31.3% (23.0–41.1)	32.9% (23.6–43.7)	.497
Diarrhea^a^	25.6% (18.5–34.4)	17.4% (11.6–25.2)	<.001
Constipation^a^	4.6% (2.6–7.9)	3.0% (1.5–6.0)	.064
Abdominal pain^a^	35.8% (22.6–51.6)	30.7% (18.4–46.6)	.036
Vomiting^a^	41.8% (30.8–53.6)	31.5% (21.6–43.4)	<.001
Nausea^a^	.6% (.2–1.5)	1.4% (.5–4.3)	.003
Headache^a^	32.5% (12.3–62.3)	33.2% (12.4–63.6)	.836
Leukopenia^a^	10.5% (5.9–17.8)	8.3% (4.1–15.8)	.254
Thrombocytopenia^a^	13.9% (7.3–25.0)	8.3% (3.8–17.1)	.009

Data are among patients with culture-confirmed enteric fever in Bangladesh, Nepal, and Pakistan.

^a^Adjusted for age.

## DISCUSSION

The diagnostic performance of clinical features , both reported symptoms and clinical signs, to distinguish enteric fever from other causes of febrile illness was moderate in this large, multi-country, multi-site study of febrile patients presenting to outpatient departments. The findings indicate that clinical presentation cannot be used to reliably screen febrile patients for further diagnostic testing. Our findings underline the need for accurate, rapid, and affordable diagnostics, particularly in low-resource settings where blood culture is typically not available.

In this study, blood culture–positive *S.* Typhi and *S.* Paratyphi A patients had, on average, a 1-degree higher temperature at presentation when compared with febrile enteric fever blood culture–negative patients; being febrile (>99.5°F) and having a high-grade fever (≥ 103°F) at presentation were predictive of both *S.* Typhi and *S.* Paratyphi A blood culture positivity. Whether this finding would hold in settings with other endemic febrile illnesses, such as malaria, warrants further exploration. Constipation and a mild cough are taught to be common in enteric fever patients [11–13]. We did find that around a third of the patients with *S.* Typhi and *S.* Paratyphi A reported a cough; however, when compared to febrile enteric fever blood culture–negative patients, the relative importance of cough diminished. On the contrary, the absence of a cough was predictive of both *S.* Typhi and *S.* Paratyphi A culture positivity. Constipation was not common in this study population. Unlike studies by Hosoglu et al. [7], Haq et al [9], Kuvandik et al [14], and Khan et al [15], we did not see an association between leukopenia and enteric fever. However, complete blood counts were not systematically collected as part of the study protocol, so this study could not systematically evaluate the predictive performance of the presence of leukopenia.

While gastrointestinal symptoms were comparable in younger age groups, adults with enteric fever were more likely to report abdominal pain, diarrhea, and vomiting than blood culture–negative febrile patients. These findings suggest that gastrointestinal symptoms among adults with 3 or more consecutive days of fever may raise the clinical suspicion of typhoid and paratyphoid.

While clinical symptoms and features, both alone and in combination, were insufficient to diagnose enteric fever, the index of suspicion should be raised in outpatient settings for patients who present with reported fever for ≥3 days, and who have one or more of the following: fever at presentation, no cough, or three or more days of being unable to conduct normal activities. These findings agree with those of Vollaard et al [8], who also reported that clinical symptoms were insufficient to diagnose enteric fever in Indonesia, and found that the absence of cough should raise clinical suspicion for the disease.

While it is classically taught that *S.* Paratyphi causes milder disease than *S.* Typhi, several recent epidemiologic studies have found no clinically distinguishing features between the 2 serovars [8, 16–18]. We found that patients with *S*. Paratyphi A presented with slightly milder symptoms, encompassing fewer days of fever, a lower temperature at presentation/admission, and fewer gastrointestinal symptoms, including abdominal pain, diarrhea and vomiting, compared with patients with *S.* Typhi. These findings contrast with those of Vollaard et al [8], Patel et al [17], and Maskey et al [18], who reported no differences in the clinical presentation of *S*. Paratyphi and *S*. Typhi. However, the Vollaard et al [8] and Patel et al [17] studies were likely underpowered to detect differences in clinical presentation, with only 92 and 82 enteric fever cases, respectively. In our study, patients with *S.* Typhi were more likely to be hospitalized than those with *S*. Paratyphi A, suggesting that the clinical syndrome of *S.* Paratyphi A is not as severe. This finding is comparable to a study in the United States, largely among returned travelers, which reported a higher percentage of hospitalizations among patients with *S*. Typhi compared to those with *S*. Paratyphi [19].

Several limitations should be considered when interpreting the results of this study. We conducted the diagnostic evaluation of clinical symptoms and features among outpatients only, and the accuracy of such symptoms and features could possibly differ in inpatient settings. However, given that the majority of enteric fever patients in this region are diagnosed in ambulatory settings, the study population is still relevant [20]. The reference standard used for the diagnostic evaluation, blood culture, is itself imperfectly valid. The sensitivity of blood culture is estimated to be 59% [6], implying that 40% of truly positive *S.* Typhi and *S.* Paratyphi A cases are missed when using this diagnostic criterion. However, we expect that the addition of missed enteric fever cases would not have had a tremendous impact on the diagnostic accuracy of the reported symptoms. For example, in a scenario where all culture-missed enteric fever cases did not have a cough, sensitivity for the absence of cough would increase from 65.5% to 78.6%. Another limitation of this study is that we did not have access to diagnostic test results for patients who were blood culture–negative for enteric fever. With this information, we could have compared the clinical features of enteric fever to alternative etiologies of fever, such as dengue and malaria. Despite these limitations, this study had several strengths. To our knowledge, it is the only multi-country study evaluating the predictive value of clinical features of enteric fever. With the large sample size and multi-country generalizability, we were able to evaluate robustly the diagnostic value of the clinical features of enteric fever.

In conclusion, our findings add support to the body of literature demonstrating that relying on clinical features and symptoms is insufficient to accurately diagnose enteric fever. The challenge of using symptoms to distinguish enteric fever from other febrile illnesses is supported by the moderate to poor diagnostic performance of clinicians' diagnoses. The results highlight the urgent need for rapid, accurate, and affordable diagnostics for enteric fever.

## Notes

***Financial support.*** This work was supported by the Bill and Melinda Gates Foundation (grant number OPP1113007).

***Supplement sponsorship.*** This supplement is sponsored by the Sabin Vaccine Institute and made possible by a grant from the Bill & Melinda Gates Foundation.
